# An application of mixed-effect models to analyse contraceptive use in Malawian women

**DOI:** 10.1186/s40834-019-0088-y

**Published:** 2019-06-27

**Authors:** Davis James Makupe, Save Kumwenda, Lawrence Kazembe

**Affiliations:** 1 University of Malawi, The Polytechnic, P/Bag 303, Chichiri, Blantyre 3, Malawi; 2 University of Malawi, Chancellor College, P.O.Box 280, Zomba, Malawi; 3 University of Namibia, Statistics and Population Studies, P/Bag 13301, Windhoek, Namibia

**Keywords:** Bayesian, Contraceptive use, Heterogeneity, Mixed Effects, Multilevel Models, Random Effects, Intra-cluster Correlation

## Abstract

In Malawi, the current approach to family planning using contraceptive methods is individualised, yet studies have shown that variability in contraceptive-use still remains after accounting for it at individual and household levels. Therefore, this study assessed variability at higher levels such as enumeration areas, districts and regions. Biasness of the estimates was addressed by the use of Bayesian approach.

The study used 2015–16 Malawi Demographic Health Survey women data. After ascertaining the significance of association of all explanatory variables with contraceptive use, the top-down (backward) stepwise model selection method was followed in the Bayesian framework using Markov Chain Monte Carlo and defuse priors. Models were compared on the basis of Deviance Information Criteria and significance of parameter estimates was checked via credible intervals while that of cross-cluster variances was checked by examining their diagnostic plots.

All the selected socio-demographic factors were strongly associated with contraceptive-use (*p*-value*<* 0.001). These factors include; region, place-of-residence, age, parity, education, occupation, marital-status and religion. It was also found that about 15 and 2.3% of the variation in contraceptive-use was attributed to enumeration area and district clustering, respectively. The single-level model underestimated the parameter estimates by at least 4% for both models. And parity-enumeration area, age-enumeration area and age-district random effects were significant in their respective models. It was also noted that most young women aged between 15 and 24 years were not using any contraceptive methods.

The study indicated that there exist significant enumeration area and district heterogeneity on contraceptive use in Malawian women and that random-effect models are the most appropriate models other than single-level models. Thus family planning programs focusing on contraceptive-use should switch to inclusive approach and statistical analyses should consider including enumeration area and district heterogeneity while controlling for the above significant factors. Stakeholders may also consider encouraging young women to use contraceptive methods, if Malawi is to minimize problems due to overpopulation.

## Introduction

Contraceptive use (CU) is pivotal to protecting women’s health and rights, influencing upon fertility and population growth and promoting economic development in developing countries [1]. Globally, contraceptives help prevent an estimated 2.7 million infant death and loss of 60 million years of healthy life [1]. However, the use of modern contraceptive methods has been low in sub-Saharan Africa, though there is evidence of an increase with time and in many developing countries there are geographical variations in CU [2, 3].

Malawi population is growing rapidly [4, 5]. The recent Malawi Population and Housing Census (MPHC) conducted in 2018 indicates that Malawi population still remains youthful with about 51% being below the age of 18 [4]. This carries a demographic momentum toward further population growth [5]. In its family planning programs, Malawi government emphasises on contraceptive methods as a means to reduced population growth rate [6].

Given Malawi’s rapid population growth, many studies have been conducted on CU to see if any improvement can be done to detour population growth. However, most of these studies focused on determinants at individual and household levels, yet previous studies suggested that variations in CU still remain after accounting for individual and household variability [2]. Additionally, multilevel modelers have statistically shown that ignoring clustering when it exists (by estimating naive classical linear model) yields biased standard error estimates. Estimated standard errors are too small (underestimated), leading to large test statistics, inflating type I error and hence spurious significant parameter estimates [7–9].

This study therefore, aimed at establishing the existence and extent of cross cluster heterogeneity at different geographical area levels in CU for Malawian women, so that stakeholders are advised on how to successfully combat overpopulation using CU. It was assumed that women are clustered within their EAs, districts or regions, since these areas have been shown to significantly affect CU due to social or physical enablers or barriers to the access of contraceptive services, which may include religion and cultural beliefs, mountains and availability of social service structures [10–12]. It was also expected that by addressing clustering in CU, significance of parameter estimates could be correctly determined. Biasness of parameters was further addressed by the use of Bayesian approach, since frequentist likelihood-based estimates tend to be biased as compared to Bayesian estimates especially where random effects are included [13–16].

## Background

The high rate of population growth and its adverse impact on the economy, environment and developmental strategies in Malawi has long been recognised. To attain Millennium Development Goals (MDGs), especially MDG 5, the government introduced family planning (FP) program as early as 1964 and adopted a National Population policy which was aimed at reducing population growth rate [6]. Due to resistance from citizens this program was discontinued. In 1982, the Malawi government, after acknowledging the health problems a woman faced when pregnancies were too early, too many, too late and too frequent, re-introduced FP program in the name of ‘national Child-Spacing’ as a part of the maternal and child health program [17]. The introduction of multiparty system in 1994, greatly improved the environment in which FP programs could be implemented. Until now, FP services can be easily accessed and more than 97% of Malawians can name at least one contraceptive method [5]. The priority of FP programs is to increase the use of effective contraceptives and improve coverage and supply strategies [18, 19]. Owing to these efforts, there have been an increase in contraceptive prevalence rate (CPR) among women from 7% in 1992 to 26% in 2000, 28% in 2004, 42% in 2010 and 58% in 2016, which is expected to rise further with current FP programs being implemented [6, 17]. This is followed by a reduction in the country’s total fertility rate [5].

Despite all these efforts and achievements in FP, the country’s population continues to grow rapidly. The total population increased by 35% from 13029498 in 2008 to 17563749 in 2018 representing a growth rate of 2.9% per annum [4]. Note that overpopulation has adverse consequences such as poverty, high childhood mortality rates, natural disasters, health problems, malnutrition, unemployment and scramble for education [20, 21].

### Contraceptive use in sub-Saharan Africa

In general, sub-Saharan African (SSA) countries have registered lower CPR in the past few years as compared to other regions in Africa and other continents such as Asia, Europe and America though the trend shows an increase with time [2, 3]. A lot of studies have shown that there are significant geographical variations in contraceptive use among women aged 15 - 49 years [1, 2, 10–12]. In a study done in 6 SSA countries (Kenya, Malawi, Tanzania, Burkina Faso, Ghana and Ivory Coast) in 2007, by Rob Stephenson and his colleagues, it was found that CPRs were high in the northern Malawi, southern Tanzania and central Kenya [2]. This clustering suggests that there might be geographical characteristics common to these regions which acted to shape contraceptive use. Community, regional or geographical variations were also observed in Ethiopia, Mali and Bangladesh by three different country-specific studies [1, 10, 11]. In Bangladesh, there were significant slum variations which were explained by slum- level variables. Ferede (2013) concluded that in Ethiopia, researchers should use multilevel models than traditional regression methods when their data structure is hierarchical as with Demographic Health Survey (DHS) data [1]. And Khan et al (2011) noted that standard logistic model seriously biases the parameter estimates when analysing multilevel data sets [12].

In many developing countries in SSA, studies have been conducted to identify the causes of the said significant geographical variations using multilevel modeling by assessing cluster-level variables. Stephenson et al in their 6 country study mentioned earlier, examined the association of contraceptive use and several contextual community-level variables, including; community-level cultural beliefs, presence of health services and routes, dominant religion in the community, mean female years of education, female and partner approval of FP and mean household amenities index. They found that CU were generally seen in wealthier households and that in Malawi Muslims were less likely to use modern contraceptives than Catholics [2].

There are many individual and household level variables that have been shown by several studies to be highly associated with CU in Malawi and SSA region. Chintsanya in his study done in Malawi, considered demographic variables such as age, place of residence, parity, ideal number of children and fertility preferences and socioeconomic factors such as education, wealth index and access to media. The aim was to compare the effects of these factors on CU across DHS conducted in 2000, 2004 and 2010 [17]. In yet another study in Malawi, Palamuleni found that use of contraceptives increased with age, parity and education. He also noted that CPR is always high in the northern region, followed by central region and lower in the southern region in 2000 and 2004 MDHS.

## Methods

This was an analytical study which aimed at quantifying the relationship between variables and random effects on CU among reproductive women in Malawi. Two-level Generalised Mixed Effect Models (GLMM) with logit link were employed to secondary data; the 2015-16 MDHS women data, considering individual women as level-1 units and EAs, districts or regions as level-2 units. The aim was to find out whether there were significant differences between EAs, districts or regions in CU and develop a model to use when analysing CU in Malawi.

### Data source

The 2015-16 MDHS whose data was used in this study was implemented by National Statistical Office (NSO) of Malawi in collaboration with the Ministry of Health (MoH) and Community Health Services Unit (CHSU). The sampling frame used for this survey was the 2008 Malawi Population and Housing Census (PHC) which was provided by NSO. The sample was designed to provide population and health indicators at the national, regional and district levels. It was selected using a stratified two-stage cluster sampling design, where EAs also referred to as clusters were the sampling units for the first stage and households comprised the second stage of sampling units. This is the reason why the study employed multilevel models. EAs which were used in the 2015-16 MDHS were made during the 2008 PHC by NSO by subdividing each district where each EA was wholly classified as urban or rural. The 2015-16 MDHS sampled 850 EAs; 173 in urban areas and 677 in rural areas. A list of households was compiled for each cluster to serve as the sampling frame for selection of households. A fixed number of 30 households per urban cluster and 33 per rural cluster were randomly selected using systematic sampling, which led to a representative sample of 27,516 households, of which 26,564 were occupied. Out of these, 26,361 were successfully interviewed (response rate of 99%). In the interviewed households 25146 eligible women were identified for individual interviews, of which 98% (24,562 women) were successfully interviewed [6].

### Variables

The response variable was the binary ‘contraceptive-use’ coded; ‘0’ for non- users and ‘1’ for users. The explanatory variables were selected for inclusion in the analysis based on their significance in various previous studies on CU [2, 5, 17, 18, 22]. The factors whose relationship with CU was examined in this study included; region (northern, central, southern), place-of-residence (urban, rural), age (15-24, 25-34, 35+), parity (no child, children (1-3), children (4+)), education (no education, primary, secondary, tertiary), occupation (not working, manual, agriculture, sales, office), marital-status (never married, widowed/divorced, separated, married), religion (Christians, Muslim, no religion, others).

### Analysis

The analysis started with descriptive statistics where each variable was summarised. Thereafter, bivariate analysis was carried out to assess the association of each factor with CU, using the chi-square test of association, so that only those factors whose categories significantly differed in CU were included in the multivariate logistic mixed-effect models. Then the crude odds-ratios (ORs) for CU comparing women in the various levels of the factors were calculated to aid comparison of this data and findings from other studies. Multilevel modeling technique was adopted because the 2015-16 MDHS data used in this study was assumed to be hierarchical, since it was collected using stratified two-stage sampling using EAs as clusters [23]. Therefore, a two-level model was adopted, considering individual women as level-1 units and EAs as level-2 units. However, it was also noted that EAs used in 2015-16 MDHS by design were nested in districts and administratively, districts are nested in regions. Consequently, districts and regions were considered as higher level clustering variables. Moreover, it is expected that different levels of geographical areas such as EAs, districts and regions can have different impact on the interest and behaviour of units within them, owing to variability in social or physical enablers or barriers to the access of FP services. Therefore, it was also imperative to explore levels of heterogeneity between districts and regions.

Though with this structure, higher level models could be possible, we considered EAs, districts and regions as all level-2 clustering factors, since we were only interested in existence of heterogeneity across these units. Therefore, the following general two-level random-effect logistic regression model as presented by many authors [7–9, 24, 26] was considered:

$$ logit\left({\pi}_{ij}\right)={\beta}_{00}+{\beta}_{10}{X}_{1 ij}+\dots +{\beta}_{p0}{X}_{pij}+{\beta}_{01}{Z}_{ij}+\dots +{\beta}_{0q}{Z}_{qj}+{\beta}_{11}{X}_{1 ij}{Z}_{1j}+\dots +{\beta}_{1q}{X}_{1 ij}{Z}_{qj}+\dots +{\beta}_{pq}{X}_{pij}{Z}_{qj}+{u}_{0j}+{u}_{1j}{X}_{1 ij}+\dots +{u}_{pj}{X}_{pij} $$

Or by using summation notation, the equation becomes [9];

$$ logit\left({\pi}_{ij}\right)={\beta}_{00}+\sum \limits_p{\beta}_{p0}{X}_{pij}+\sum \limits_q{\beta}_{0q}{Z}_{qj}+\sum \limits_p\sum \limits_q{\beta}_{pq}{X}_{pij}{Z}_{qj}+\sum \limits_p{u}_{pj}{X}_{pij}+{u}_{0j} $$

In this model, *logit*(*π*_*ij*_) = ln (*π*_*ij*_*/* (1 *π*_*ij*_)) is log-odds for contraceptive-use called ‘the logit link’, chosen because we were interested, not only in the probability of the success, but also a comparison of probabilities of success in two different groups [25]. The symbol, *π*_*ij*_ is a probability of CU for a woman, *i* in any EA, district or region, *j*. In the linear predictor (the right hand side of the equation), *X*s are level-1 predictors and *Z*s are level-2 predictors. The assumption is that we have *P* level-1 predictors and *Q* level-2 predictors indicated by the subscripts *p* (1*, . . ., P)* and *q* (1*, . . ., Q*), respectively. The subscript *j* is for the cluster (*j* = 1*, . . ., J*) i.e. EAs, districts or region while the subscript *i* is for individual women (*i* = 1*, . . ., n*_*j*_). *β*s are fixed coefficients for the *P* level-1 predictors, the *Q* level-2 predictors and the *P* × *Q* interaction terms between level-1 and level-2 predictors (*X*_*pij*_*Z*_*qj*_). For a logistic model *β*s are log-odds which by exponentiation we obtain odds ratios. *u* are random error terms across clusters, *j* for all (*P* + 1) parameter estimates. The assumption here is that the effects of all level-1 variables vary across clusters due to level-2 variables. However, it should be noted that level-1 error term, *ϵ* is not included in the model since the model regresses a transformed mean for non-normal (binary) response variable and the error-term is part of the specification of the error distribution [7, 9, 15, 25]

The GLMM analysis process was stepwise. The first step examined the null (intercept only) model. Thereafter, the correct model selection protocol presented and recommended by many authors [9, 26] was followed to identify a significant model for CU in Malawi. This protocol, usually referred to as ‘the top-down strategy’ [26], presents a backward stepwise model selection procedure. It starts with a full model with as many predictors as possible and their interactions, including many random effects as it can be theorised. Then by comparison of models, insignificant components of the random part are dropped and then the significance of the components of the fixed part is checked [26, 27]. Usually if the opposite direction is taken, the required information may end up in the random effects, as a result some of the important predictors may be dropped [26]. We then firstly optimised the random part and then the fixed part. To avoid over-parameterisation, random effects were induced on few level- 1 variables which were either covariates or factors with at most two categories. Over-parameterisation leads to inconsistency of the variance parameters, long time for parameter estimation and non-convergence of parameter estimates in Bayesian framework [26, 28].

For a start, a full (co)variance matrix was assumed, where variances of all random errors at level-2 and their covariances were estimated. To check the significance of each component, a component was dropped and the model was compared to the one with that component using Deviance Information Criteria (DIC). Convergence of the MCMC iterations was checked by means of MCMC summary (diagnostic) plot. Significance of the estimates of the fixed effects was checked via credible intervals. In this study, all the variables were considered to be measured at individual level except for the factors; ‘region’ and ‘place-of-residence’ which were assumed to have been measured at district or EA levels. A model with no level-2 variables but random effects both on the intercept and slopes of all variables is known as a ‘random coefficient model’ [9]. It is usually referred to as ‘the mixed-effect model’ if some slopes are allowed to vary across clusters while some are fixed across clusters. If only random intercept is significant and estimated, the model is called ‘the random intercept model’ and has the form;

$$ lo\mathrm{g} it\left({\pi}_{ij}\right)={\beta}_{00}+{\beta}_1{X}_{1 ij}+\dots +{\beta}_p{X}_{pij}+{u}_{0j}. $$

Random intercepts are used to model unobserved heterogeneity in the overall response and random coefficients model unobserved heterogeneity in the effects of explanatory variables on the response [11].

The null model also known as ‘empty’, ‘variance component (VC)’ or ‘intercept only’ model for a 2-stage logistic regression has the following form;

$$ logit\left({\pi}_{ij}\right)={\beta}_{00}+{u}_{0j} $$

The model helps in determining and assessing the intra-class correlation (ICC) which is the proportion of the variance explained by the grouping structure found by taking a ratio of variance at cluster level to the total variation [9, 27]. The formula for ICC in the 2-stage logistic empty model is as follows;

$$ ICC=\frac{\sigma_{u0}^2}{\sigma_{u0}^2+\frac{\pi^2}{3}} $$

where *σ*^2^is the variance of level-2 error term and *π*^2^*/*3 represents the variance of individual level error term.

Finally, the classical linear regression model was compared to the optional EA and district random effect models to appreciate the importance of the EA and district random effects.

## Results

### Prevalence of contraceptive use

Table 1 shows prevalence of CU among women in their respective groups and presents results from bivariate analysis. Out of 24562 women who were interviewed, 11194 (45.6%) said they had ever used some contraceptive methods to delay pregnancy. Comparatively, prevalence of CU was higher in the central region (47.2%) and in the rural areas (46.1%). It was also higher in middle aged (between 25 and 34 years) (58.1%), women with 4 or more children (61.7%) married and business women (59.1% and 56.4%, respectively) and women with lower education levels (more than 47% of women with primary education and those with no education). Very low prevalence rates were observed among women with no children (6.3%), unmarried women (10.0%) and young women aged between 14 and 24 (30.7%).

**Table 1:** CU prevalence and bivariate analysis between CU and individual- and cluster-level factors

Characteristic	*n*	% CU	*p*	OR	CRI
Overall	24,562	45.57			
Region			*<* 0*.*001		
Northern	4803	45.14		1	
Central	8417	47.15		1.085	(1.014, 1.167)
Southern	11,342	44.59		0.908	(0.908, 1.050)
Place-of-residence			*<* 0*.*001		
Urban	5247	43.49		1	
Rural	19,315	46.14		1.114	(1.048, 1.184)
Age(years)			*<* 0*.*001		
15–24	10,367	30.74		1	
25–34	7624	58.12		3.126	(2.939, 3.333)
35+	6571	54.42		2.690	(2.531, 2.886)
Parity			*<* 0*.*001		
No child	5782	6.30		1	
Children(1–3)	11,307	55.02		18.247	(16.200, 20.430)
Children(4+)	7473	61.68		23.975	(21.242, 26.924)
Education			*<* 0*.*001		
No education	2779	47.46		1	
Primary	15,028	47.56		1.003	(0.925,1.091)
Secondary	6061	40.52		0.753	(0.688,0.825)
Tertiary	694	39.05		0.707	(0.598,0.848)
Occupation			*<* 0*.*001		
Not working	8422	34.27		1	
Manual work	4037	52.02		2.080	(1.928, 2.241)
Agriculture	9374	50.78		1.983	(1.876, 2.099)
Business	1135	56.39		2.481	(2.182, 2.808)
Office	1594	50.69		1.979	(1.773, 2.232)
Marital-Status			*<* 0*.*001		
Never married	5326	9.99		1	
Divorced/widowed	1979	36.43		5.165	(4518, 5.847)
Separation	1305	38.85		5.726	(4.953, 6.586)
Married	15,952	59.14		13.066	(11.870,14.454)
Religion			*<* 0*.*001		
Christians	21,685	46.61		1	
Muslim	2726	37.42		0.685	(0.631, 0.742)
No religion	113	45.13		0.946	(0.661, 1.359)
Others	38	42.11		0.825	(0.426, 1.619)

The odds for CU for a woman living in the central region were 1.09 and 1.19 (=1.085/0.908) compared to the northern and southern region, respectively (Table 1). This means that women in the central region were 9% and 19% more likely to use contraceptives than northern and southern region women, respectively. Similarly, rural women had 11% higher odds for CU than urban women. Middle aged and older women were 213% and 169% more likely to use contraceptives than younger women, while women with no children were 95% and 96% less likely to use contraceptive methods as compared to women with one to three children and those with four or more children, respectively. Women with higher education (secondary and tertiary) were more than 30% less likely to use contraceptive methods as compared to women with no education. Business and office women were 148% and 98% while married women were 1207% more likely to use contraceptives than unemployed and unmarried women, respectively. The odds for CU for Muslim women were 0.69 times those of Christian women. That is, Muslim women had about 31% lowered odds for CU as compared to Christian women.

### Random-effect models for contraceptive-use

#### Null models

The results for the three null models for random effects, assessing heterogeneity between clusters and dependence of individual women on EAs, district and region are presented in Table 2.

**Table 2:** EA, district and regional random effect null models

Clustering Variable	Mean (95% CRI)	*σ*_*u*_(95% CRI)	ICC	DIC
EA	− 0.198 (− 0.251, − 0.152)	0.567 (0.039,1.392)	0.147	33,644.76
District	−0.218 (− 0.354, − 0.089)	0.078 (0.029,0.160)	0.023	34,033.69
Region	−0.194 (− 0.419, − 0.036)	0.027 (0.000,0.078)	0.008	34,331.37

Using DICs, it was noted that the model with EA heterogeneity had a smaller DIC (DIC = 33,644.76) as compared to the ones which assumed district variability (DIC = 34,033.69) and region variability (DIC = 34,331.37). This means that the model with EA variations fitted the data relatively well as compared to the other two null models. Similarly, the model with district heterogeneity fitted the data better than the one with regional heterogeneity.

**Fig. 1:**
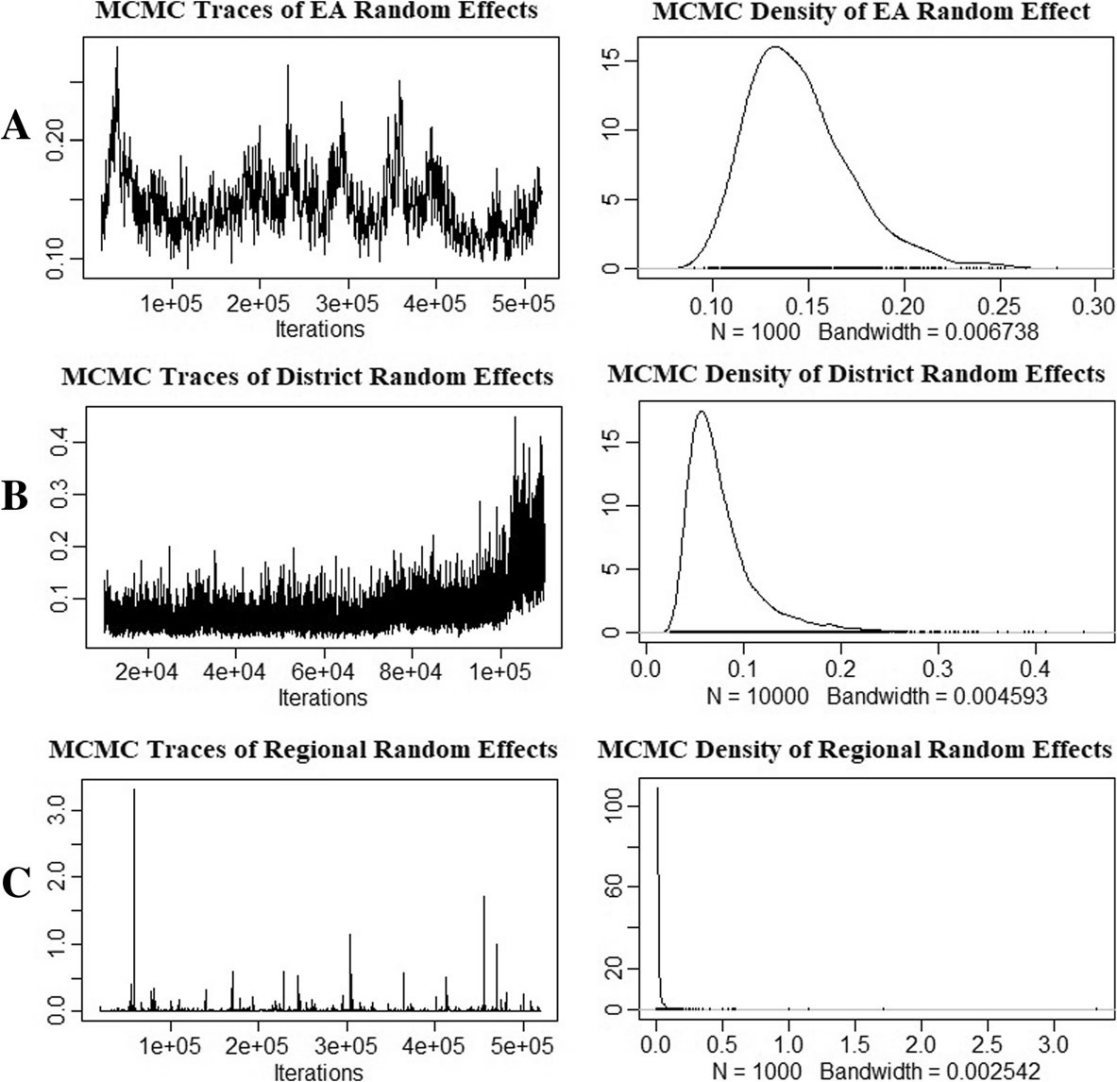
Diagnostic plots of cross-cluster variances for EA, district and regional heterogeneity null models

Considering the cross-cluster variances, it was noted that cross-EA variance (*σ*_*u*_ = 0*.*567) was larger than the cross-district variance (*σ*_*u*_ = 0*.*078) and cross-region variance (*σ*_*u*_ = 0*.*027). This means that there was substantial cross-EA heterogeneity as compared to cross-district and cross-region heterogeneity. Furthermore, though it is not advisable to use credible intervals for variances, since they are always positive [26], but the credible interval (CRI) for the cross-region variance is entirely close to zero. This indicates that most iterations produced zero variance estimates. Figure 1 shows the diagnostic plots (i.e. posterior MCMC traces and their probability distributions) for the cross-cluster variances for the three models.

Figure 1a and b are MCMC traces and posterior density distributions of cross-EA and cross-district variance. The density distributions show that the iterations were normally distributed though slightly right skewed. Figure 1c shows diagnostic plots of cross-region variance. It is clear from this figure that iterations converged to zero, except for some few unusually big iteration values (outliers). Therefore, we valued EA and district clustering, since they were shown to be valid by their diagnostic plots and CRIs.

It was also noted that there were differences between the crude odds-ratio (OR) calculated as 0.837 (=11,194/13,368) and the ones in the EA variance component model (OR=0.820=*e*^*−*0*.*198^) and district variance component model (OR = 0.804= *e*^*−*0*.*218^). They both adjusted slightly lower from the crude odds-ratio, meaning that the EA and district variance component model explained some variations in CU. The odds-ratio for the regional variance component model (OR = 0.824) was very close to the crude odds-ratio of 0.837 as compared to the odds-ratio for EA and district variance component models. Thus regional variance component model explained very little variations on CU.

The corresponding intra-EA and intra-district correlations (ICCs) were found to be 0.147 and 0.023, respectively. The ICC values mean that the correlation between individuals in the same EA or district on decisions regarding CU was 0.147 or 0.023, respectively i.e. about 15% of variations in CU among Malawian women was attributed to EA clustering and 2.3% to district clustering. Note that it is indicated that the reported ICCs for districts can be as small as 0.01 [29]. Therefore, we assumed that both EA and district ICC were large enough and worth accounting for.

#### Model selection

At this stage two different models were considered and optimised concurrently; one with EA-random effects and the other with district-random effects. We started with the full conditional parametric mixed-effect models. In the EA-random effect model, intercept, parity and age effects were assumed variable across EAs due to regional and place-of-residence influences while in the district-random effect model, it was assumed that in additional to the three effects, place-of-residence also varied across districts with regional influences. In both models, we assumed unstructured (co)variance matrix to model correlation of cluster-specific parameter estimates. The DICs for the full EA and district mixed-effect models estimated in this step were 27,582.03 and 27,750.11, respectively (Table 3).

**Table 3:** Comparison of DICs assessing significance of random effects on EA and district random effect models

Model	DIC	*χ* ^*2*^	*df*	*p*-value
EA Random effect models				
Full model	27,582.03			
No parity random effects	27,625.85	43.82	3	*<* 0.001
No age random effects	27,602.48	20.45	3	*<* 0.001
No covariances	27,583.33	1.30	3	0.729
No interaction terms	27,588.26	4.93	6	0.553
District random effect models				
Full model	27,750.11			
No parity random effects	27,757.08	6.97	4	0.138
No age random effects	27,770.25	20.14	4	0.001
No POR random effects	27,751.81	1.70	4	0.791
Age random effects only	27,760.64	10.53	7	0.160
No covariances	27,759.73	9.62	8	0.293
No interaction terms	27,757.26	7.15	14	0.929

Following backward stepwise protocol, random effects were fixed one-by-one to assess their randomness across clusters. The first part in Table 3 shows EA random effect models. Two models; one without parity-EA random effects and the other without age-EA random effects were compared to the full EA random effect model. It was observed that both parity-EA random effects and age-EA random effects were significant (*p <* 0*.*001), and hence they were maintained in the model. This means that parity and age effects on CU were significantly different across EAs. However, comparing the full model i.e. the model with both parity-EA and age-EA random effects and the one that dropped covariances between random intercepts and parity-EA random effects, random intercept and age-EA random effects and parity-EA random effects and age-EA random effects, it was noted that these three covariances were collectively insignificant (*χ*^2^ = 1*.*30*, df* = 3*, p* = 0*.*729), hence they were dropped. This means that there was no correlation between variances across EA. Therefore, the model that was adopted at this stage was a mixed-effect model with random intercept, parity-EA and age-EA random effects but without covariances between these random effects.

The full district random effect model assumed random intercept, parity, age and place-of-residence random effect. Comparing this model and each of the three models which dropped one of the three random effects (parity, age and place-of-residence), it was found that parity-district random effects and place- of-residence-district random effects were insignificant (*p* = 0*.*138 and *p* = 0*.*791, respectively), while age random effects were significant (*χ*^2^ = 20*.*14*, df* = 4*, p* = 0*.*001). Furthermore, the model with random intercept and age-district random effects indicated that parity-district and place-of-residence-district random effects were collectively insignificant when compared to the full model. Therefore, these two random effects were not considered in the subsequent models. In the next model, as with EA random effect models, convariance between random intercept and age-district random effects was dropped. Comparing this model and the full model, it was noted that this covariance, parity-district and place-of-residence random effects were collectively insignificant, hence they were dropped. Therefore, at this stage we ended up with the model with random intercept and age random effect without covariance between these random effects.

We then checked the relevance of the interaction terms in both models. The EA random effect model includes regional and place-of-residence interactions with the two variables which were assumed to be random (i.e. parity and age). The model which dropped all the interaction terms gave a smaller DIC (DIC = 27,588.26) as compared to the full model (DIC = 27,582.03). Therefore, the interaction terms were collectively considered insignificant. This means EA’s region or it’s place-of-residence did not affect parity and age EA specific effects on CU. Similarly, with district random effect model which assumed that the random effects were due to whether a district is in the northern, central or southern region, the model without regional interaction terms was estimated. Comparing this model and the model with age-district random effects (which dropped parity-district and place-of-residence-district random effects) without a covariance term between age random effects and random intercept, it was noted that this model had a smaller DIC (DIC = 27,757.26) while the later had a larger DIC (DIC = 27,759.73). This indicated that the interaction terms were also insignificant here. Thus the model without regional interaction terms fitted the data well than the one with interaction terms. This means that a district’s region did not affect parity, age and place-of-residence effects on the woman’s decision to use contraceptives.

Significance of the fixed components was verified in a similar manner. Variables were dropped one-by-one and comparison of the models was accomplished by the use of DIC. In both models it was realised that all predictors individually and collectively assisted in explaining variability in CU in Malawi (*p <* 0*.*001), hence they were maintained in both models. These variables included; place-of- residence, parity, age, region, education, occupation, marital-status and religion.

### Mixed-effect models compared to single level model

Now, suppose that the EA and district heterogeneity is ignored and instead a classical (single-level) logistic regression model is estimated. Table 4 presents the log-odds for both EA random effect model (EAREM) and district random effect model (DREM), found optimal in this study, alongside the single-level logistic regression model (SLM) for comparison.

**Table 4:** Parameter estimates of EA- and district-random effect models and classical single-level regression model

Parameter	Mixed effect model	Classical linear model	
	EAREM	DREM	SLM
Fixed effects			
Intercept	−3.962	− 3.817	−3.300
Parity	0.451	0.417	0.344
Age	− 0.059	−0.056	− 0.045
Place-of-residence(urban)			
Rural	−0.211	-0.208	−0.167
Region (northern)			
Central	0.214	0.157	0.189
Southern	0.164	0.099	0.140
Education (no education)			
Primary	0.381	0.360	0.369
Secondary	0.635	0.611	0.587
Secondary	0.704	0.666	0.620
Occupation (not working)			
Manual	0.473	0.453	0.421
Agriculture	0.368	0.316	0.295
Business	0.661	0.667	0.564
Office	0.467	0.433	0.397
Marital-Status (never married)			
Widowed/divorced	1.434	1.479	1.251
Separation	1.562	1.579	1.342
Married	2.594	2.597	2.175
Religion (Christian)			
Muslim	−0.521	−0.349	−0.544
Other	−0.442	− 0.445	− 0.362
Religions no religion	− 0.264	− 0.215	− 0.171
Random effects			
${\sigma}_{u(intercept)}^2$	0.120	0.086	
${\sigma}_{u(age)}^2$	0.001	0.0005	
${\sigma}_{u(parity)}^2$	0.011		

*EAREM* Enumeration-Area random effect model, *DREM* District random effect model, *SLM* Single-level model

There were considerable differences between parameter estimates of SLM and each of the two random effect models. Generally, SLM underestimates the parameter estimates. The higher amount of underestimation was observed on age effect (31% for EAREM and 24% for DREM). The understanding is that the differences in parameter estimates were a result of the inclusion of EA or district random effects in the two random effect models. In fact, these were the components which were making these models different, otherwise they would have been the same model. This implies that the random effects helped to explain substantial variability which was not accounted for in the single-level model. Therefore, it can be said that SLM was inappropriate for analysis of CU in Malawi. Thus mixed-effect models were favored against the single-level model.

## Discussion

The results have shown that cross-EA and cross-district heterogeneity do exist in the CU data in Malawi. The random effect models indicated that the crude odds comparing contraceptive users and non-users significantly vary from one EA to another and from one district to another. Furthermore, it was noted that the models with cross-EA and cross-district variations fitted the data better than the one with cross-regional variations. It was indicated that there existed considerable amount of EA and district heterogeneity worth accounting for in the CU data in Malawi. These findings are consistent with findings from other studies. For instance, one of the studies in sub-Saharan Africa found that developing countries (such as Malawi) have substantial geographical variations in CU, although the factors shaping these variations are little understood [2]. And in another related study in Bangladesh, it was reported that there was slum-level variability in CU which was explained by slum-level variables [11]. Therefore, it was also hoped that there must be some EA- and district-level factors contributing to these variations which are yet to be identified. These factors may include some social or physical enablers or barriers to the access of contraceptive methods such as road network and presence of social structures like hospitals or some contextual factors such as proportion of women exposed to family planning massages and mean number of births [10]. On the other hand, it was found that regional heterogeneity was insignificant in this study, though a study in Ethiopia found significant regional variability [1]. Perhaps this was because Ethiopia has a large number of regions (11 regions) which may be considered to be robust enough as opposed to only three regions in Malawi. This implies that EA and district random effects, but not regional random effects, should be considered when analysing CU in Malawi, otherwise the standard errors of the parameter estimates are bound to be biased leading to unreliable parameter estimates [12, 27].

The final EA random effect model where all selected, significant women-level variables were controlled for, indicated that the adjusted overall odds comparing users and non-users (the intercept), parity and age effects were significantly random between EAs. It was also found that intercept and age effect were random across districts. This means that random effect model should be considered when analysing CU in Malawian women by allowing the intercept and the effects of some variables such as parity and age to vary across EAs and districts. Separate studies conducted in Ethiopia and Mali, supported random intercept models for CU [1, 10]. It was also noted that while all the components added to the models significantly explained substantial variability in CU, some variations still remain unexplained (the intercepts were still significant in the final models). It was expected that these remaining EA and district heterogeneity would be explained by EA or district level variables [12, 29].

From the bivariate analysis it was found that all eight selected socio-demographic factors were individually significantly associated by CU. These factors include; region, parity, age, place-of-residence, education, occupation, marital-status and religion. This is what was expected, for these factors were selected because they were shown to be significant in some previous studies [2, 5, 11, 17]. However, it was noted that young women aged between 15 and 24 are less likely to use contraceptive methods. This is contrary to what many studies reported [5, 11, 30]. Robey et al reported that CU increases in young women and declines in older women [30].

In this study we employed Bayesian approach and multilevel analysis on the hierarchical MDHS data that was collected using multistage sampling. Therefore, we are confident that the findings are not heavily biased. They should be valid and reliable.

However, we did not consider many possible contextual EA or district level variables which could potentially explain variability in CU between EAs and districts. This means that we are unable to identify the causes of these variations.

## Conclusion

In this study, all eight above mentioned socio-demographic factors were significantly associated with CU. Therefore, studies on CU in Malawi should consider adjusting for these factors.

It was also found that EA and district heterogeneity were significant and important in the analysis of CU. This means that women in the same EA or district influence each other and make similar decision pertaining to CU while women in different EAs or districts differ in their decision. Therefore, random-effect models should be prioritised when analysing CU in Malawi by considering EAs and districts as clusters. On the other hand, EAs and districts with lower CU prevalence can be identified and targeted in the subsequent interventions to improve their CU prevalence rate and that Government and other stakeholders should consider inclusive approach by considering EAs and districts as units other than sticking to the current individualised approach. For instance, some women in the EAs or districts can be trained to act as mentors to their fellows in the same EA or district or health experts in FP can be deployed to EAs or districts to render FP services and motivate women as with what was done in agriculture and health sector in the past years.

However, the study did not consider many of the possible (structural or contextual) EA and district level variables which potentially could help identify the sources of variability in CU between EAs and districts [2, 10]. Therefore, we recommend further studies on the sources of EA and district heterogeneity which can assess the effects of EA and district level variables. Furthermore, models with more levels which consider women as being nested in EAs and EAs in districts and those that incorporate spacial variations (such as spacial and geo-additive models) can be considered.

The findings indicate that region, parity, age, place-of-residence, education, occupation, marital-status and religion are important predictors for CU. Therefore, when conducting studies on CU, statisticians and analysts should continue considering these factors. However, it was observed that most young women do not use contraceptive methods. This is a potential threat to population that can lead to overpopulation in the near future. Therefore, government and other stakeholders in health sector should consider programs that can encourage them to use contraceptive methods.
